# The Relative Importance of Vascular Risk Factors on Early Cognitive Aging Varies Only Slightly Between Men and Women

**DOI:** 10.3389/fnagi.2022.804842

**Published:** 2022-03-28

**Authors:** Nadine Bonberg, Niklas Wulms, Klaus Berger, Heike Minnerup

**Affiliations:** Institute of Epidemiology and Social Medicine, University of Münster, Münster, Germany

**Keywords:** sex, cognitive aging, cardiovascular risk factors, bias, susceptibility

## Abstract

**Objective:**

To investigate the sex-specific course and impact of vascular risk factors on cognitive aging in a rather young and healthy community-dwelling cohort.

**Methods:**

We used data from a population-based cohort study, collected three times during 6 years, comprising 1,911 examinations from 798 participants aged 35–66 years at baseline. Cognitive performance on the Color-Word-Interference-Test, the Trail Making Tests (TMT) A&B, the Word Fluency Test, a 12-item word list, the Purdue Pegboard Test and a principal component global score were used as outcomes in linear mixed models. We evaluated (1) sex differences in cognitive trajectories, (2) the mediating role of hypertension, diabetes, smoking and obesity [body mass index (BMI) > 30] on sex differences and (3) in sex-stratified analyses, potential sex-specific effects of these risk factors on cognition.

**Results:**

For all cognitive tests, we observed cognitive decline with age. Rates of decline slightly differed across sexes, showing a later but steeper decline for women in tests of memory (word list) and word fluency, but a steeper decline for men in tests of psychomotor speed and mental set shifting (TMT A&B) in older age. Women generally scored better on cognitive tests, but the slightly higher prevalence of classical vascular risks factors in men in our cohort could not explain these sex differences. Sex-stratified analyses revealed a generally small, concordantly negative, but quantitatively slightly different impact of diabetes, smoking and obesity on cognitive functions but mixed effects for arterial hypertension, depending on the blood pressure values, the treatment status and the duration of arterial hypertension.

**Conclusion:**

Cognitive sex differences in this rather young and healthy cohort could not be explained by a differing prevalence of vascular risks factors across sexes. The association of cardiovascular risk factors with cognition, however, slightly differed between men and women, whereby effects were generally small. Whereas longtime diabetes, obesity and smoking had a sex-specific, but concordantly negative impact on psychomotor speed, executive and motor functions, we found some opposing effects for arterial hypertension. Our results can help to identify sex-specific susceptibilities to modifiable risk factors, to attract attention to potential information bias and to stimulate further research into alternative causes and mechanism of sex differences in cognitive aging.

## Introduction

Sex differences in cognition have been observed over many domains and populations (McCarrey et al., 2016; Reas et al., 2017; Fu et al., 2020; Levine et al., 2021; Nichols et al., 2021). Most studies in high-income countries show that men outperform women in some spatial tasks, while women usually outperform men in most other domains, particularly verbal tasks (McCarrey et al., 2016; Reas et al., 2017). However, the causes for this sex difference are not fully revealed yet and are presumably multifactorial (Levine et al., 2021; Nichols et al., 2021). Besides biological reasons, such as differences in brain reserve, hormone profiles and the prevalence of potentially brain-damaging risk factors, several environmental (e.g., education and socioeconomic factors) as well methodological factors (e.g., age and selection of the population), have to be acknowledged in the analysis and interpretation of cognitive sex differences (Reas et al., 2017; Volgman et al., 2019; Bloomberg et al., 2021; Nichols et al., 2021; van Zutphen et al., 2021). When examining cognitive aging, i.e., the trajectories of cognitive performance over time, even more challenges arise, such as selective attrition and test-retest effects (Salthouse, 2019; Rouanet et al., 2021).

Regarding modifiable risk factors, it is established that cardiovascular risk factors are associated with vascular and degenerative brain damage and an increased risk of cognitive decline and dementia (Debette et al., 2011; Gorelick et al., 2011; Gottesman et al., 2017; Boots et al., 2019; Livingston et al., 2020). Arterial hypertension and diabetes mellitus, particularly when acquired in midlife, are probably the single most important adversaries of cognitive aging, having been consistently associated with declines in executive functions, attention, memory as well as processing and motor speed (Biessels et al., 2008; Monette et al., 2014; Gottesman et al., 2017; Biessels and Despa, 2018; Iadecola and Gottesman, 2019). Besides, systemic low-grade inflammation, obesity and smoking have been identified as risk factors for cognitive decline, though the evidence is less consistent (Beeri et al., 2009; Hajjar et al., 2018; Zheng and Xie, 2018; Vintimilla et al., 2019; Walker et al., 2019).

The question that arises from this evidence on sex differences on the one hand, and vascular risk factors, on the other hand, is, whether the former can at least partially be explained by the latter in the context of cognitive aging. It is known that men and women have different vascular risk profiles. For example, the midlife prevalence of classical vascular risk factors in women is generally lower compared to men of the same age and socio-economic background (Lerner and Kannel, 1986), and women differ from men with regard to hormonal and inflammatory status (Khera et al., 2005; Lakoski et al., 2006).

Besides differences in the prevalence of vascular and metabolic risk factors, men and women might differ regarding the susceptibility to the damage these risk factors potentially cause in the brain. Studies on this aspect are rather rare, but there is recent evidence that suggests that specific risk factors, such as smoking and diabetes, might have a differential impact on men and women and thus might further explain some of the sex differences in cognitive aging (Appelman et al., 2015; Biessels and Despa, 2018; van Zutphen et al., 2021).

Based on 6-year longitudinal data from the population-based cohort of the BiDirect Study, the goal of the current analysis was to reveal sex-specific cognitive trajectories for a variety of distinct neuropsychological tests and to evaluate the role of cardiovascular risk factors on the specific test performances. We also examined the effect of potential biases, such as test-retest effects and selective attrition, in the association between risk factors and cognitive aging.

## Materials and Methods

### Study Population

The BiDirect Study is a cohort study conducted in Münster, Germany (Teismann et al., 2014). The primary aim of the study is to investigate the bidirectional relationship between depression and (subclinical) atherosclerosis. It is based on the examination of three distinct cohorts comprising (1) patients with an acute episode of depression, (2) patients with a recent cardiovascular event and (3) population-based controls, who had been randomly recruited by use of the population register of the city of Münster. In the current analysis, three examinations of the population-based control participants were used. The baseline examination of 911 population-based controls aged 35–66 years took place between 2010 and 2013, 800 participants returned for a second examination between 2013 and 2016 after a mean follow-up time of 2.7 years and 680 for the third examination between 2016 and 2018 after a mean follow-up time of another 2.7 years. At all examinations, participants underwent a computer-guided interview, self-administered questionnaires, sensory and neuropsychological assessments, clinical examinations (e.g., anthropometry, vascular status and blood sampling), as well as magnetic resonance imaging of the brain (Teismann et al., 2014; Teuber et al., 2017). The data acquisition was conducted by a trained study team. For the current analyses, we applied several exclusion criteria. We excluded participants with neurological disorders and limited German language skills, as well participants with missing or invalid neuropsychological test results resulting in a total of 798 out of 911 participants in our analysis. This study was approved by the Ethics Committee of the University of Münster and the Westphalian Chamber of Physicians in Münster, North-Rhine-Westphalia, Germany. All participants gave their written informed consent for study participation.

### Assessment of Sociodemographic and Health Status

Smoking status, socio-demographic characteristics and data on participants’ health status and histories, such as physician’s diagnosis of diabetes and hypertension, were assessed in a personal interview at baseline and follow-ups. Education was documented in the four categories (1) primary or general secondary school, (2) intermediate secondary school, (3) high school and (4) university graduates. Current medications were denoted and blood pressure as well as body weight and height were measured in a standardized way (Teismann et al., 2014). For analysis, a categorical variable “arterial hypertension” was defined as a combination of current hypertensive treatment (yes/no) and measured blood pressure (controlled/uncontrolled) and therefore labeled as (1) untreated, controlled, (2) untreated, uncontrolled, (3) treated, controlled and (4) treated, uncontrolled blood pressure. Uncontrolled blood pressure was defined as a systolic blood pressure of at least 140 mmHg and/or a diastolic blood pressure of at least 90 mmHg. For sensitivity analysis, we used another definition of hypertension given as physician’s diagnosis (no diagnosis, diagnosed ≤ 10 years, diagnosed > 10 years). A history of diabetes was classified into “no physician diagnosis,” “diagnosed ≤ 7 years” and “diagnosed > 7 years.” Body mass index (BMI) was calculated from measured weight and height (kg/m^2^) and categorized into no obesity (BMI < 30 kg/m^2^) and obesity (BMI ≥ 30 kg/m^2^). Smoking status was defined categorically as never vs. former vs. current smoking. Self-reported depressive symptoms were assessed by the Center for Epidemiological Studies Depression-Scale (CES-D) at baseline and follow-up examinations (Teismann et al., 2014). A CES-D score ≥ 16 was used to define clinically relevant depressive symptoms (Radloff, 1977).

### Neuropsychological Assessment

Five validated tests were administered to all study participants at BiDirect baseline and follow-ups (Teismann et al., 2014).

(1) *Color–Word Interference Test (CWIT)*: Participants performed a paper-pencil version with three task sets (words, color, color–word) with 36 items each. The reaction time was measured for each task set (Stroop, 1935). We focused on the second and third condition (color and color–word) and calculated the time difference of these conditions (interference time) to measure interference control, a measure of working memory capacity.

(2) *Trail Making Test (TMT) A&B*: In TMT A participants were asked to connect consecutive numbers from 1 to 25 as fast as possible to measure attention and psychomotor speed. In TMT B, they have to connect consecutive numbers and letters in an alternating sequence (1-A-2-B-3-C, etc.) to measure working memory and mental set shifting (Reitan, 1992). The time needed to complete each part was recorded.

(3) *Regensburg Word Fluency Test (“animal naming test”)*: Participants were asked to name as many animals as possible within 60 s to measure categorical association (semantic) fluency as a measure or executive function (Morris et al., 1989; Tombaugh et al., 1999).

(4) *Word List*: To measure verbal retentiveness and memory, a recorded 12-item emotional word memory list was presented via loudspeaker to the participants (Kissler et al., 2006). After the presentation of the word list, the participants were asked to reproduce as many words as possible. A second presentation of the word list with immediate recall followed. After these two presentations, a third free recall followed after an interval of 15 min.

(5*) Purdue Pegboard Test*: Participants were asked to place as many pegs as possible into a wooden board within 30 s, first with the right hand, followed by the left hand, to measure fine motor skills (Tiffin and Asher, 1948).

We calculated a Z-score for each test or subtest result using the respective test mean and standard deviation (*SD*) of the female baseline control group for standardization. Test results from TMT A and B as well as the reaction times and the interference time from CWIT were log-transformed before standardization. All Z-scores were scaled in a way, that higher values represent better results. Afterwards, we averaged the scores for the three runs of the word list and the scores for the right and left hand from the Purdue Pegboard Test.

#### Assessment of a Global Cognitive Score

We made a principal component analysis with baseline data (Z-scores from word list, Pegboard, interference time from CWIT, TMT A&B, word fluency test) by using the R-package “psych” [function principal ()] (Revelle, 2021) and extracted one component as a global score. Based on the estimates of this analysis, we calculated the global score for the follow-ups and standardized the score with the mean and SD of the female baseline values.

### Statistical Analyses

We first present the cognitive trajectories for men and women. After these descriptive analyses, we test for a mediating role of cardiovascular risk factors in the association between sex and cognitive performance. Finally, using sex-stratified analyses we estimate the individual effects of several vascular risk factors on cognition for men and women separately. All statistical analyses were performed with R 4.1.0 (R Core Team, 2021) and RStudio Version 1.4.1717 (RStudio Team, 2021). We used the lmer function from the R-package lme4 (Bates et al., 2015) for linear mixed models and produced plots with the R-package interactions (Long, 2019). Analyses were conducted with a 2-tailed alpha of 0.05 referred as a statistically significant level.

#### Trajectories of Standardized Neuropsychological Test Results

We assessed trajectories for the different standardized neuropsychological test results using linear mixed models with random intercepts. In each model, age at study participation was used as a time variable. To account for possible nonlinear trends, we included age as a natural spline with two degrees of freedom (df) in our models. Additionally, sex and interaction of sex and age (as spline) were included, because we expected different slopes for men and women. Furthermore, we added the “number of study participations” to account for possible practice effects. In the model for memory (word list), we included the interaction of age (as spline) and the number of study participations to allow for a variation of the practice effect with age. All models were adjusted for potential selective attrition (see below).

#### Adjustment for Selective Attrition

To account for potential sex differences in outcome-related dropouts (i.e., study dropouts due to impaired cognition) we used inverse probability weighting (IPW) in our models (Rouanet et al., 2021). We calculated probabilities for study participation at the two follow-ups with logistic regression models. These logistic regression models comprise age, sex, sociodemographic variables, distance to study center and variables describing the health condition in prior surveys, including the most recent cognitive global score. The inverse values of these probabilities were used as weights in the mixed models described above. Further information on IPW can be found here (Seaman and White, 2013; Hernán and Robins, 2020). As sensitivity analyses, we re-analyzed all models using the same cohort without IPW.

#### The Mediating Role of Cardiovascular Risk Factors in the Association Between Sex and Cognitive Performance

Weighted linear mixed models were used to assess the association of sex with standardized neuropsychological test results. We performed two models with different adjustments for every neuropsychological test. In addition to sex, models of type one comprise age (spline, 2 df), number of study participations and education. Models of type two were additionally adjusted for hypertension, diabetes, CES-D score, smoking status and obesity. Models for memory (word list) additionally include the interaction of age and number of study participations, and models for the Purdue Pegboard Test include body height as a proxy for hand size. IPW was used to account for potential selective attrition (see above).

#### Sex-Specific Effects of Risk Factors on Cognition

We built weighted linear mixed regression models with IPW for the standardized neuropsychological test results stratified by sex and added age (spline, 2 df), education and the number of study participations (and for memory the interaction of these), diabetes, CES-D score, obesity, smoking status and hypertension as explaining variables. In the models for the Purdue Pegboard Test, we also added body height as a proxy for hand size.

## Results

We used longitudinal data (baseline and two follow-ups) consisting of 1,911 examinations from 798 participants of the BiDirect study for analysis. Table 1 shows the characteristics of the study population at baseline and follow-ups. At baseline, 47% of participants were male. Median age at baseline was 53 years (range: 35–66 years), at first follow-up 57 years (range: 37–69) and at second follow-up 60 years (range: 40–71 years). In all, 76 (22%) men and 82 (21%) women were current smokers at baseline and 137 men (39%) and 218 women (55%) had untreated and controlled blood pressure, while 115 men (33%) and 88 women (22%) had an untreated and uncontrolled, 31 (9%) men and 38 (10%) women a treated and controlled and 66 (19%) men and 50 (13%) women a treated and uncontrolled blood pressure. A CES-D score of at least 16 was documented for 47 (13%) men and 84 (21%) women at baseline.

**TABLE 1:** Study population at baseline and two follow-ups.

	Baseline		First follow-up		Second follow-up	
	Men *N* = 349	Women *N* = 394	Men *N* = 311	Women *N* = 361	Men *N* = 228	Women *N* = 268
**Age (years)**						
(Median, range)	53 (35–66)	54 (35–66)	56 (38–69)	57 (37–69)	59 (40–71)	61 (40–71)
**Education, *N* (%)**						
Primary or general secondary school	74 (21%)	68 (17%)	62 (20%)	61 (17%)	37 (16%)	48 (18%)
Intermediate secondary school	48 (14%)	112 (28%)	40 (13%)	98 (27%)	32 (14%)	63 (24%)
High school	63 (18%)	75 (19%)	51 (16%)	71 (20%)	36 (16%)	53 (20%)
University graduates	164 (47%)	139 (35%)	158 (51%)	131 (36%)	123 (54%)	104 (39%)
**Diabetes, *N* (%)**						
No	335 (96%)	380 (96%)	292 (94%)	346 (96%)	216 (95%)	259 (97%)
0-7 years	7 (2%)	7 (2%)	11 (4%)	9 (2%)	4 (2%)	5 (2%)
> 7 years	7 (2%)	7 (2%)	8 (3%)	6 (2%)	8 (4%)	4 (1%)
**Hypertension, *N* (%)**						
Untreated, controlled	137 (39%)	218 (55%)	121 (39%)	198 (55%)	93 (41%)	148 (55%)
Untreated, uncontrolled	115 (33%)	88 (22%)	88 (28%)	73 (20%)	45 (20%)	50 (19%)
Treated, controlled	31 (9%)	38 (10%)	48 (15%)	43 (12%)	57 (25%)	40 (15%)
Treated, uncontrolled	66 (19%)	50 (13%)	54 (17%)	47 (13%)	33 (14%)	30 (11%)
**CES-D score, *N* (%)**						
< 16	302 (87%)	310 (79%)	279 (90%)	294 (81%)	207 (91%)	229 (85%)
≥ 16	47 (13%)	84 (21%)	32 (10%)	67 (19%)	21 (9%)	39 (15%)
**Smoking status, *N* (%)**						
Never	140 (40%)	164 (42%)	116 (37%)	146 (40%)	79 (35%)	114 (43%)
Former	133 (38%)	148 (38%)	131 (42%)	153 (42%)	106 (46%)	117 (44%)
Current	76 (22%)	82 (21%)	64 (21%)	62 (17%)	43 (19%)	37 (14%)
**Obesity, *N* (%)**						
No (BMI < 30 kg/m^2^)	270 (77%)	315 (80%)	243 (78%)	297 (82%)	176 (78%)	220 (82%)
Yes (BMI ≥ 30 kg/m^2^)	79 (23%)	79 (20%)	68 (22%)	64 (18%)	52 (22%)	48 (18%)

### Trajectories of Standardized Neuropsychological Test Results

**FIGURE 1:**
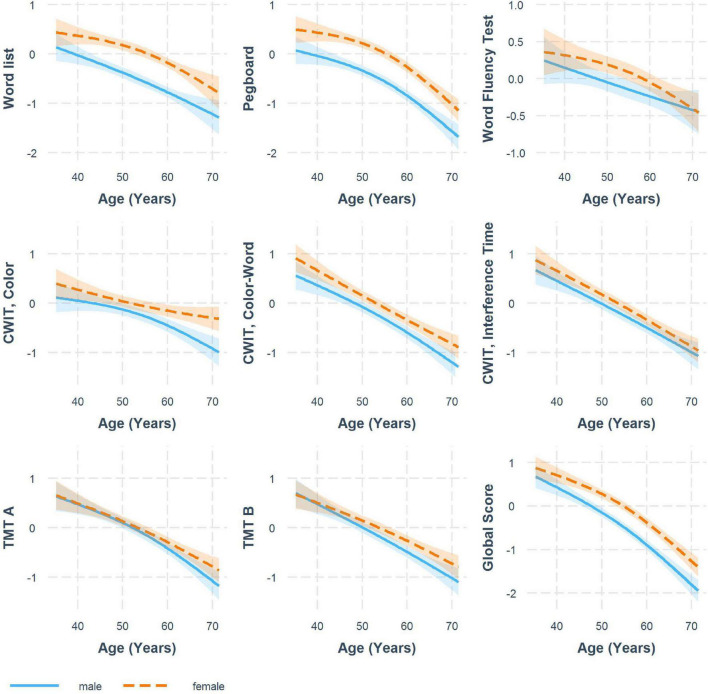
Comparison of sex-specific trajectories for standardized neuropsychological test results shown at first study participation with 95% confidence intervals (CI). Results from weighted linear mixed models.

Figure 1 shows trajectories for the standardized cognitive test results at first study participation as results of weighted linear mixed regression models. For all cognitive tests, we observed a cognitive decline with age. Descriptively viewed, women, on average, outperformed men, especially in memory (word list), the Pegboard Test, the Word Fluency Test (at least in some age regions) and the global score. Trajectories for the different conditions of the CWIT showed a lesser superiority of women and the TMTs showed only a slight superiority of women at later ages. Also descriptively, the trajectories for women showed a later, but steeper cognitive decline in tests of memory (word list), word fluency and the global score, but steeper declines for men in tests of psychomotor speed and mental set shifting (TMT A&B) in older age.

### The Mediating Role of Cardiovascular Risk Factors in the Association Between Sex and Cognitive Performance

Significant sex differences were found before and after further adjustments for vascular risk factors (Table 2). Adjusted for education, number of study participations and age, female sex was positively associated with memory [$\hat{\beta }$ = 0.57, 95% CI = (0.47, 0.67)], Pegboard [0.39, (0.24, 0.54)], interference time of CWIT [0.18, (0.07, 0.30)], TMT B [0.20, (0.08, 0.32)], Word Fluency Test [0.21, (0.09, 0.33)] and the global score [0.48, (0.36, 0.59)]. Additional adjustments for smoking status, obesity, hypertension, CES-D score and diabetes resulted in significant positive and only slightly lower associations of female sex with the cognitive test results (Table 2).

**TABLE 2:** Association of sex with neuropsychological test results (z-scores) with and without adjustment for cardiovascular risk factors.

	Word list*^c^*	Pegboard*^d^*	CWIT (interference time)	TMT A	TMT B	Word fluency test	Global score
	$\hat{\beta }$ (95% CI)	$\hat{\beta }$ (95% CI)	$\hat{\beta }$ (95% CI)	$\hat{\beta }$ (95% CI)	$\hat{\beta }$ (95% CI)	$\hat{\beta }$ (95% CI)	$\hat{\beta }$ (95% CI)
**Models 1*^a^***							
**Sex (Ref.: male)**							
Female	0.57***	0.39***	0.18**	0.10	0.20***	0.21***	0.48***
	(0.47, 0.67)	(0.24, 0.54)	(0.07, 0.30)	(−0.01, 0.22)	(0.08, 0.32)	(0.09, 0.33)	(0.36, 0.59)
**Models 2*^b^***							
**Sex (Ref.: male)**							
Female	0.56***	0.37***	0.16**	0.11	0.20**	0.20**	0.46***
	(0.46, 0.66)	(0.22, 0.52)	(0.05, 0.28)	(−0.01, 0.22)	(0.08, 0.31)	(0.08, 0.33)	(0.35, 0.57)
**Diabetes (Ref: no)**							
0−7 years	0.07	−0.09	−0.10	−0.20	−0.01	−0.03	−0.04
	(−0.16, 0.29)	(−0.33, 0.16)	(−0.37, 0.17)	(−0.48, 0.08)	(−0.27, 0.24)	(−0.32, 0.25)	(−0.25, 0.17)
> 7 years	0.02	−0.61***	−0.54**	−0.37*	−0.42*	−0.32	−0.53***
	(−0.26, 0.30)	(−0.91, −0.30)	(−0.87, −0.21)	(−0.70, −0.03)	(−0.75, −0.10)	(−0.67, 0.04)	(−0.81, −0.25)
**Hypertension (Ref: untreated, controlled**)							
Untreated, uncontrolled	−0.05	0.02	−0.11*	−0.001	−0.04	0.01	−0.05
	(−0.13, 0.03)	(−0.07, 0.11)	(−0.21, −0.01)	(−0.10, 0.10)	(−0.13, 0.05)	(−0.09, 0.12)	(−0.12, 0.02)
Treated, controlled	−0.05	0.08	−0.02	0.003	−0.12	0.06	−0.04
	(−0.16, 0.07)	(−0.04, 0.21)	(−0.15, 0.12)	(−0.14, 0.14)	(−0.25, 0.01)	(−0.08, 0.20)	(−0.14, 0.07)
Treated, uncontrolled	−0.07	−0.01	0.01	−0.001	0.003	−0.09	−0.05
	(−0.19, 0.04)	(−0.14, 0.11)	(−0.13, 0.14)	(−0.14, 0.14)	(−0.13, 0.14)	(−0.23, 0.06)	(−0.16, 0.06)
**Smoking (Ref.: never)**							
Former	−0.02	−0.22***	−0.09	−0.03	−0.04	−0.01	−0.11*
	(−0.12, 0.07)	(−0.33, −0.12)	(−0.21, 0.02)	(−0.14, 0.09)	(−0.16, 0.07)	(−0.14, 0.11)	(−0.21, −0.01)
Current	−0.07	−0.35***	−0.07	−0.12	−0.18*	−0.12	−0.20**
	(−0.19, 0.05)	(−0.48, −0.22)	(−0.21, 0.07)	(−0.26, 0.02)	(−0.32, −0.04)	(−0.27, 0.03)	(−0.32, −0.07)
**Obesity (Ref: no)**							
Yes	−0.06	−0.10	−0.13*	−0.04	−0.10	−0.07	−0.13**
	(−0.16, 0.04)	(−0.21, 0.01)	(−0.25, −0.004)	(−0.17, 0.08)	(−0.21, 0.02)	(−0.20, 0.06)	(−0.24, −0.03)

Marginal R-squared models 2	0.30	0.28	0.19	0.15	0.20	0.12	0.40
Conditional R-squared models 2	0.71	0.69	0.62	0.58	0.68	0.62	0.85

*p < 0.05, **p < 0.01, ***p < 0.001.

*^a^Adjusted for age (spline, 2 df), number of study participations and education.*

*^b^Adjusted for age (spline, 2 df), number of study participations, education and CES-D score.*

*^c^Word List: averaged z-scores for the three runs of the Word List. Results are additionally adjusted for the interaction of age and number of study participations.*

*^d^Pegboard: averaged z-scores for the right and left hand from Purdue Pegboard Test.*

*Results are additionally adjusted for body height. Results from weighted linear mixed models with 1,911 examinations from 798 participants.*

### Practice Effects

**FIGURE 2:**
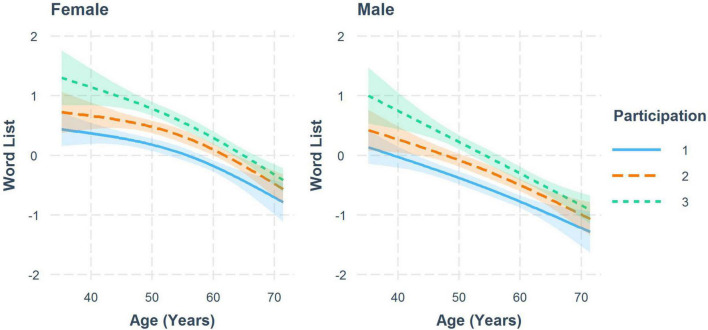
Trajectories for standardized test results of the word list by number of study participations to visualize the practice effect for women and men with 95% CI. Results from a weighted linear mixed model.

Sex-specific trajectories for memory (word list) at all three study participations are shown in Figure 2. A decline with age can be observed for men and women for all participations, as well as a distinct practice effect, that slightly decreases with age. Smaller practice effects could be also observed for the interference time of CWIT, TMT A, TMT B (women), Word Fluency Test (women) and the global score (Table 3B).

**TABLE 3A:** Sex-specific effects of potential risk factors on standardized test results from the word list and Purdue pegboard test.

	Word list*^a^*		Pegboard*^b^*	
	Men	Women	Men	Women
	$\hat{\beta }$ (95% CI)	$\hat{\beta }$ (95% CI)	$\hat{\beta }$ (95% CI)	$\hat{\beta }$ (95% CI)
**Diabetes (Ref: no)**				
0-7 years	0.09	−0.004	−0.27	0.05
	(−0.24, 0.43)	(−0.31, 0.30)	(−0.63, 0.09)	(−0.29, 0.39)
> 7 years	−0.01	0.04	−0.91***	−0.28
	(−0.41, 0.40)	(−0.35, 0.43)	(−1.34, −0.48)	(−0.71, 0.14)
**Hypertension (Ref: untreated, controlled)**				
Untreated, uncontrolled	−0.07	−0.04	0.03	−0.01
	(−0.19, 0.04)	(−0.16, 0.07)	(−0.09, 0.16)	(−0.14, 0.11)
Treated, controlled	0.04	−0.13	0.18*	−0.01
	(−0.13, 0.21)	(−0.28, 0.02)	(0.004, 0.36)	(−0.18, 0.15)
Treated, uncontrolled	−0.09	−0.05	0.04	−0.09
	(−0.26, 0.07)	(−0.21, 0.12)	(−0.13, 0.22)	(−0.27, 0.09)
**Smoking (Ref.: never)**				
Former	−0.09	0.05	−0.19*	−0.22**
	(−0.25, 0.06)	(−0.08, 0.18)	(−0.35, −0.03)	(−0.36, −0.08)
Current	−0.11	−0.02	−0.36***	−0.30***
	(−0.29, 0.07)	(−0.18, 0.14)	(−0.55, −0.18)	(−0.48, −0.12)
**Obesity (Ref: no)**				
Yes	−0.04	−0.07	−0.07	−0.11
	(−0.18, 0.11)	(−0.21, 0.07)	(−0.22, 0.09)	(−0.26, 0.05)

Marginal R-squared	0.26	0.26	0.28	0.24
Conditional R-squared	0.71	0.66	0.69	0.65

*p < 0.05, *p < 0.01, ***p < 0.001. Weighted linear mixed models with 888 examinations from 373 men and 1,023 examinations from 425 women, respectively, adjusted for education, CES-D score, age (natural spline, df = 2), number of study participations, the interaction of age and number of study participations (word list), and body height (pegboard).

*^a^Averaged z-scores for the three runs of the Word List Test.*

*^b^Averaged z-scores for the right and left hand from Purdue Pegboard Test.*

*Results from linear mixed models stratified by sex.*

**TABLE 3B:** Sex-specific effects of risk factors on standardized test results of Color–Word Interference Test (CWIT, interference time), Trail Making Test (TMT) A, TMT B, Word Fluency and the PCA-derived global score.

	CWIT (interference time)		TMT A		TMT B		Word fluency test		Global score	
	Men	Women	Men	Women	Men	Women	Men	Women	Men	Women
	$\hat{\beta }$ (95% CI)	$\hat{\beta }$ (95% CI)	$\hat{\beta }$ (95% CI)	$\hat{\beta }$ (95% CI)	$\hat{\beta }$ (95% CI)	$\hat{\beta }$ (95% CI)	$\hat{\beta }$ (95% CI)	$\hat{\beta }$ (95% CI)	$\hat{\beta }$ (95% CI)	$\hat{\beta }$ (95% CI)
**Study participation (Ref: first)**										
Second	0.26***	0.17***	0.01	0.05	0.02	0.08	0.04	0.19***	0.16***	0.22***
	(0.16, 0.36)	(0.08, 0.27)	(−0.09, 0.11)	(−0.05, 0.14)	(−0.07, 0.11)	(−0.01, 0.17)	(−0.06, 0.14)	(0.09, 0.29)	(0.09, 0.23)	(0.15, 0.28)
Third	0.36***	0.37***	0.14*	0.14*	0.05	0.14*	−0.02	0.24***	0.23***	0.38***
	(0.24, 0.48)	(0.25, 0.48)	(0.01, 0.27)	(0.03, 0.26)	(−0.07, 0.16)	(0.03, 0.24)	(−0.14, 0.11)	(0.12, 0.36)	(0.13, 0.32)	(0.29, 0.46)
**Diabetes (Ref: no)**										
0−7 years	−0.09	−0.12	−0.46*	0.07	−0.07	0.02	−0.22	0.08	−0.27	0.14
	(−0.48, 0.31)	(−0.50, 0.25)	(−0.86, −0.06)	(−0.31, 0.46)	(−0.45, 0.30)	(−0.34, 0.38)	(−0.63, 0.19)	(−0.31, 0.47)	(−0.58, 0.04)	(−0.14, 0.42)
> 7 years	−0.73**	−0.27	−0.67**	−0.05	−0.41	−0.43	−0.49	−0.23	−0.76***	−0.32
	(−1.20, −0.25)	(−0.74, 0.20)	(−1.15, −0.20)	(−0.53, 0.43)	(−0.87, 0.04)	(−0.89, 0.03)	(−0.99, 0.01)	(−0.73, 0.27)	(−1.17, −0.35)	(−0.70, 0.06)
**Hypertension (Ref: untreated, controlled)**										
Untreated, uncontrolled	−0.13	−0.10	−0.04	0.02	0.002	−0.08	−0.03	0.07	−0.08	−0.03
	(−0.27, 0.004)	(−0.23, 0.04)	(−0.18, 0.10)	(−0.13, 0.16)	(−0.13, 0.13)	(−0.21, 0.05)	(−0.18, 0.11)	(−0.08, 0.21)	(−0.18, 0.02)	(−0.13, 0.07)
Treated, controlled	−0.11	0.08	0.11	−0.09	0.03	−0.25**	0.16	−0.01	0.07	−0.12
	(−0.30, 0.09)	(−0.11, 0.26)	(−0.09, 0.32)	(−0.28, 0.10)	(−0.15, 0.22)	(−0.43, −0.07)	(−0.05, 0.36)	(−0.20, 0.19)	(−0.08, 0.23)	(−0.26, 0.02)
Treated, uncontrolled	−0.04	0.07	−0.01	0.01	0.10	−0.07	−0.08	−0.07	−0.04	−0.05
	(−0.23, 0.16)	(−0.13, 0.27)	(−0.21, 0.19)	(−0.20, 0.22)	(−0.09, 0.28)	(−0.27, 0.12)	(−0.28, 0.13)	(−0.28, 0.14)	(−0.19, 0.11)	(−0.21, 0.10)
**Smoking (Ref.: never)**										
Former	−0.09	−0.08	−0.02	−0.01	−0.10	0.02	−0.07	0.02	−0.11	−0.07
	(−0.26, 0.09)	(−0.23, 0.07)	(−0.19, 0.16)	(−0.17, 0.15)	(−0.27, 0.07)	(−0.14, 0.17)	(−0.25, 0.12)	(−0.15, 0.18)	(−0.26, 0.04)	(−0.21, 0.06)
Current	0.05	−0.19*	−0.08	−0.14	−0.19	−0.15	−0.22	−0.04	−0.16	−0.21*
	(−0.15, 0.26)	(−0.38, −0.001)	(−0.28, 0.13)	(−0.34, 0.06)	(−0.39, 0.01)	(−0.35, 0.04)	(−0.43, 0.001)	(−0.25, 0.16)	(−0.33, 0.02)	(−0.37, −0.04)
**Obesity (Ref: no)**										
Yes	−0.08	−0.16	−0.06	−0.04	−0.15	−0.03	−0.03	−0.11	−0.11	−0.15*
	(−0.26, 0.09)	(−0.33, 0.01)	(−0.23, 0.12)	(−0.22, 0.13)	(−0.32, 0.01)	(−0.19, 0.14)	(−0.21, 0.16)	(−0.29, 0.07)	(−0.25, 0.04)	(−0.29, −0.01)

Marginal R-squared	0.20	0.19	0.19	0.13	0.22	0.18	0.09	0.15	0.38	0.39
Conditional R-squared	0.66	0.58	0.62	0.56	0.70	0.67	0.64	0.60	0.86	0.83

*p < 0.05, **p < 0.01, ***p < 0.001.

*Weighted linear mixed models with 888 examinations from 373 men and 1,023 examinations from 425 women, respectively, adjusted for age (natural spline, df = 2), education and CES-D score. PCA, principal component analysis.*

*Results from linear mixed models stratified by sex.*

### Sex-Specific Effects of Risk Factors on Cognition

The sex-specific impact of vascular risk factors on standardized cognitive test results are shown in Tables 3A, 3B. Diabetes ≤ 7 years was negatively associated with TMT A in men [−0.46 (−0.86, −0.06)]. Diabetes > 7 years was negatively associated with Pegboard in men (−0.91 [−1.34, −0.48]), interference time of CWIT in men [−0.73, (−1.20, −0.25)], TMT A in men [−0.67, (−1.25, −0.20)] and the global score in men [−0.76, (−1.17, −0.35)]. A treated and controlled blood pressure was positively associated with Pegboard in men [0.18, (0.004, 0.36)] and negatively associated with TMT B in women (−0.25, [−0.43, −0.07]). As a sensitivity analysis, we performed the same analysis but used the physician’s diagnosis to define hypertension (Supplementary Tables 1A,B). A diagnosis of hypertension > 10 years was positively associated with Pegboard in men [0.26, (0.05, 0.47)]. No further significant results for this definition of hypertension were found. Smoking was negatively associated with Pegboard in men [former: −0.19, (−0.35, −0.03), current: −0.36, (−0.55, −0.18)] and women [former: −0.22, (−0.36, −0.08), current: −0.30, (−0.48, −0.12)], the interference time of CWIT in women [current: −0.19, (−0.38, −0.001)] and with the global score in women [current: −0.21, (−0.37, −0.04)]. Obesity was negatively associated with the global score in women [−0.15, (−0.29, −0.01)]. Of the potential confounding variables, higher education was positively associated with all cognitive tests apart from Pegboard (women) where no significant association was found. Moreover, a CES-D score ≥ 16 was negatively associated with Pegboard in men [−0.17, (−0.33, −0.02)] and women [−0.16, (−0.29, −0.04)], TMT A in women [−0.22, (−0.36, −0.08)] and TMT B in men [−0.29, (−0.46, −0.11)].

### Sensitivity Analyses

For all above-mentioned analyses, the re-analyses on the unweighted data showed similar results (data not shown).

## Discussion

The presented work investigated sex differences in early cognitive aging from multiple perspectives. First, a descriptive approach presents sex-specific trajectories for various cognitive functions. Second, the influence of vascular risk factors on cognitive sex differences is elucidated by investigating their mediating role as well as differing susceptibilities across men and women. Third, the role of selective attrition and information bias is investigated.

### Cognitive Trajectories

Using a large population-based cohort, we were able to show sex-specific trajectories for a large battery of neuropsychological tests reflecting a broad spectrum of cognitive abilities. As expected, and adding to the growing body of evidence, we generally found cognitive decline with age in our cohort (Reas et al., 2017; van Zutphen et al., 2021). We also observed that women, except for the TMT A, outperformed men for most age ranges. We observed the most pronounced effect of sex in the reproduction of the 12-item word list (short-term memory) with an effect size of ß = 0.57 (adjusted for age, education and study participation). Our findings here corroborate and expand prior work on mostly older cohorts that also reported sex differences in tasks of memory, executive function and attention (Van der Elst et al., 2006; McCarrey et al., 2016; Reas et al., 2017; Luck et al., 2018; Fu et al., 2020; Levine et al., 2021; Nichols et al., 2021; van Zutphen et al., 2021). Regarding sex differences of the Purdue Pegboard Test in a normal aging population, there are only few and conflicting reports (Peters et al., 1990; Schmidt et al., 2000; Sivagnanasunderam et al., 2015). Though motors skills are known to diminish with age and correlate with cognitive decline (Kluger et al., 1997; Hamilton et al., 2017), to our knowledge this is the first study to show persistent sex differences in a prospective population-based study.

Though not statistically tested for, we also observed sex differences in the longitudinal rates of change, with women showing steeper declines for memory, verbal fluency and fine motor skills, beginning at the age of around 55 years (Figure 1). On tests of psychomotor speed in contrast (TMT A and B, CWIT color task), men declined faster beginning at 50 years. The evidence so far is rather inconsistent with reports of later and steeper declines in men or women (McCarrey et al., 2016; Reas et al., 2017; Bloomberg et al., 2021; Levine et al., 2021; van Zutphen et al., 2021). Reasons for these inconsistencies might lay in the use of different tests, but also in differing age periods and age cohorts under study, as well as in methodological differences regarding the modeling of cognitive decline. In general, comparable to our findings, reported sex differences in the rates of cognitive decline were generally small in other studies and mostly affected only some cognitive domains (McCarrey et al., 2016; Reas et al., 2017; Bloomberg et al., 2021; Levine et al., 2021; van Zutphen et al., 2021).

### Comparison of Risk Factor Profiles

Men, on average, were slightly better educated, showing a higher proportion of university graduates. Regarding cardiovascular risk factors, we did not observe large sex differences. Men more often reported to be active smokers, particularly during follow-up examinations. Moreover, men more often showed uncontrolled blood pressures, though the absolute percentage and the sex discrepancy diminished during follow-up. The prevalence of obesity was comparable across sexes, as was diabetes. Self-reported depressive symptoms were more pronounced in women.

Taken together, our cohort represents a rather well-educated and healthy population (Teismann et al., 2014; Schneider et al., 2021) with only small sex discrepancies in the prevalence of cardiovascular risk factors. Nevertheless, as expected and generally observed in populations from high-income countries, men showed a slightly worse cardiovascular risk factor profile, whereas women reported more depressive symptoms (Lerner and Kannel, 1986; Seedat et al., 2009). Moreover, we observed a decline in the prevalence of risk factors and depressive symptoms over time, which might be due to selective attrition (healthy study adherer). We thus used IPW to account for differential loss-to-follow-up.

### The Role of Risk Factors in the Association of Sex and Cognition

The examined risk factors explained next to nothing of the variation in cognition across sexes, as similar effect sizes of sex on cognitive performances were seen before and after adjustment in the linear mixed models (Table 2). The mere difference in the prevalence of cardiovascular risk factors across sexes thus did not explain the observed sex differences in cognition. This was expected in our cohort of relatively young and healthy participants, as there was hardly any sex difference in the distribution of vascular risk factors. Nevertheless, these findings corroborate earlier work in mainly older cohorts, that also found only small mediating effects of cardiovascular risk factors on the association between sex and cognition, suggesting that alternative biological mechanisms play a role in cognitive sex differences (Levine et al., 2021; van Zutphen et al., 2021).

Looking at the sex-stratified analyses and thus the impact of the different risk factors on cognitive test results in men and women separately, we found evidence of a slight sex-specific susceptibility: tough most of the risk factors showed a concordantly negative impact on cognitive test performance, the size of the effects slightly differed across sexes. Longtime diabetes was associated with worse performance in many cognitive processes, such as psychomotor speed and mental set switching (TMT A & B), interference control (CWIT) and fine motor functions (Pegboard), however, only in men. This agrees with findings from van Zutphen et al. (2021) who reported sex differences in the impact of diabetes on processing speed. No clear sex discrepancies emerged for former and current smoking, which were about equally associated with worse performances in the Pegboard Test, whereas there was a slightly stronger association of current smoking with the global score and the CWIT in women and the word fluency test in men. For obesity, we found an association with the global score in women only. Taken together, effect sizes differed between men and women, but they were generally small and there is no clear preference of one sex over the other sex. One can assume multiple reasons for varying susceptibilities across men and women, such as differing genetic profiles with varying gene-environment interactions or differences in structural and functional brain reserve (Marrocco and McEwen, 2016; McEwen and Milner, 2017; Levine et al., 2021). However, this is by now highly speculative and should stimulate further studies including brain imaging, hormone measurements and genetic data.

Most interestingly, for arterial hypertension we observed mixed effects on cognition for men and women. Treated and controlled arterial hypertension showed a positive association with fine motor skills in men and a negative association with TMT B in women - both compared to participants without hypertension. The exposure “arterial hypertension” is prone to misclassification due, for example, to unknown periods of undiagnosed hypertension, the impact of treatment, the age at onset, and the type of hypertension (systolic vs. diastolic). We have included the treatment status as well as the actual blood pressure in our primary definition. We also used an alternative definition defining arterial hypertension as a known physician’s diagnosis and additionally accounting for the period of known hypertension. Here again, we found a diagnosis of hypertension > 10 years to be positively associated with the Pegboard Test in men. So taken together, a long-lasting and adequately treated arterial hypertension, respectively, is associated with better motor performance even compared to normotensive men and non-diagnosed men, respectively. One potential explanation might be that this group presents a highly health-conscious sample with early and optimal interventions of not only hypertension but several vascular risk factors. Interestingly, other studies also reported positive effects of hypertension on cognition (Forte et al., 2019; van Zutphen et al., 2021), but our study also found sex differences in this association. Effects were opposite in women showing worse performance in the TMT B in women with treated and controlled arterial hypertension. There are several potential explanations for these contrasting findings across sexes, such as sex disparities in the initiation, vigor or response to antihypertensive treatment (Lefort et al., 2018; Iadecola and Gottesman, 2019; Kalibala et al., 2020), or an interaction of blood pressure or treatment with sex hormones, especially around the time of menopause (Volgman et al., 2019). All these mechanisms should be further elucidated in future studies.

### Strengths and Limitations

Several limitations must be considered in the interpretation of our data. Although we analyzed nearly 800 participants in a longitudinal design, the number of participants with risk factors, particularly diabetes was relatively low. Given the small effect of vascular risk factors and the only minor sex differences in this relatively young and healthy cohort our analyses from a post hoc view were probably underpowered to detect some statistically significant sex differences. Nevertheless, focusing on the effect sizes this does not considerably affect the interpretation that the estimates slightly varied across sexes and that a major proportion of sex differences in cognitive aging is not explained by cardiovascular risk factors in this population. As methodological challenges, we focused on potential biases like selective attrition and information bias. In this study population, not every participant participated in all surveys. That could lead to biased results due to selective loss to follow-up, particularly when dropout due to cognitive decline is different across sexes. To reduce this selection bias, we calculated models with IPW. We also performed sensitivity analyses with unweighted models and observed stable results. Thus, we can assume that selective attrition is very low in this study. Another problem was the definition of hypertension. The exposure “arterial hypertension” is generally prone to misclassification due to unknown periods of undiagnosed hypertension, the impact of treatment, the age at onset, the type of hypertension (systolic vs. diastolic) etc. We have included the treatment status as well as the actual blood pressure and additionally used a second definition using the physician’s diagnosis to define hypertension as accurate as possible. The longitudinal design by itself is a clear strength, but we had to deal with strong practice effects especially in the analysis for memory, that even dominated the age-related decline leading to successive improvements of memory performance over time. This discrepancy between cross-sectional and longitudinal age-cognition relations, especially for the cognitive domain of memory, and the inherent difficulty of avoiding or eliminating the practice effect, was also described by others (Salthouse, 2019). Other strengths of this study are the repeated risk factor and neuropsychological assessment allowing for time-dependent modeling, as well as the relatively young cohort and the use of the Pegboard Test.

## Conclusion

In conclusion, cognitive trajectories differ between men and women. However, cardiovascular risk factors seem to play only a minor role in the explanation of these sex differences in this cohort of rather healthy younger to middle-aged men and women. Whereas diabetes, smoking and obesity had a negative but quantitatively slightly different impact on psychomotor speed, executive and motor functions across sexes, we found no adverse effects of any of the risk factors on memory. For arterial hypertension, we found opposing effects on mental set shifting and motor skills across sexes. Corroborating other work, we also found significant and persistent sex differences for most cognitive tests, that could not be explained by differing risk factor profiles, which should stimulate further investigations into the development and maintenance of the brain and cognitive reserve in even younger adults or adolescents. Our results might help to classify cognitive test results and identify sex-specific susceptibilities to modifiable risk factors. On the methodological side, they might stimulate deeper investigations into the assessment and definition of risk factors, and the development of strategies to avoid or overcome the practice effects in repeated test situations.

## Data Availability Statement

The datasets presented in this article are not readily available because investigators must submit an application to access BiDirect data. Requests to access the datasets should be directed to the Institute of Epidemiology and Social Medicine, University of Münster, Germany.

## Ethics Statement

The study involving human participants were reviewed and approved by Ethics Committee of the University of Münster and the Westphalian Chamber of Physicians in Münster, North-Rhine-Westphalia, Germany. The participants provided their written informed consent to participate in this study.

## Author Contributions

NB drafted the manuscript, conducted, and programmed all statistical analyses. HM came up with the research question, supervised the analyses, and supported the drafting of the manuscript. KB and NW made substantial contributions to the data acquisition and revised the manuscript for intellectual content. All authors contributed to the article and approved the submitted version.

## Conflict of Interest

The authors declare that the research was conducted in the absence of any commercial or financial relationships that could be construed as a potential conflict of interest.

## Publisher’s Note

All claims expressed in this article are solely those of the authors and do not necessarily represent those of their affiliated organizations, or those of the publisher, the editors and the reviewers. Any product that may be evaluated in this article, or claim that may be made by its manufacturer, is not guaranteed or endorsed by the publisher.

## References

[B1] Appelman Y. van Rijn B. B. Ten Haaf M. E. Boersma E. Peters S. A. E. (2015). Sex differences in cardiovascular risk factors and disease prevention. *Atherosclerosis* 241 211–218. 10.1016/j.atherosclerosis.2015.01.027 25670232

[B2] Bates D. Mächler M. Bolker B. Walker S. (2015). Fitting linear mixed-effects models using lme4. *J. Stat. Softw.* 67 1–48. 10.18637/jss.v067.i01

[B3] Beeri M. S. Ravona-Springer R. Silverman J. M. Haroutunian V. (2009). The effects of cardiovascular risk factors on cognitive compromise. *Dialogues Clin. Neurosci.* 11 201–212. 10.31887/DCNS.2009.11.2/msbeeri 19585955 PMC3093131

[B4] Biessels G. J. Despa F. (2018). Cognitive decline and dementia in diabetes mellitus: mechanisms and clinical implications. *Nat. Rev. Endocrinol.* 14 591–604. 10.1038/s41574-018-0048-7 30022099 PMC6397437

[B5] Biessels G. J. Deary I. J. Ryan C. M. (2008). Cognition and diabetes: a lifespan perspective. *Lancet Neurol.* 7 184–190. 10.1016/S1474-4422(08)70021-8 18207116

[B6] Bloomberg M. Dugravot A. Dumurgier J. Kivimaki M. Fayosse A. Steptoe A. (2021). Sex differences and the role of education in cognitive ageing: analysis of two UK-based prospective cohort studies. *Lancet Public Health* 6 e106–e115. 10.1016/S2468-2667(20)30258-9 33516287 PMC8141610

[B7] Boots E. A. Zhan L. Dion C. Karstens A. J. Peven J. C. Ajilore O. (2019). Cardiovascular disease risk factors, tract-based structural connectomics, and cognition in older adults. *NeuroImage* 196 152–160. 10.1016/j.neuroimage.2019.04.024 30980900 PMC6713222

[B8] Debette S. Seshadri S. Beiser A. Au R. Himali J. J. Palumbo C. (2011). Midlife vascular risk factor exposure accelerates structural brain aging and cognitive decline. *Neurology* 77 461–468. 10.1212/WNL.0b013e318227b227 21810696 PMC3146307

[B9] Forte G. De Pascalis V. Favieri F. Casagrande M. (2019). Effects of blood pressure on cognitive performance: a systematic review. *J. Clin. Med.* 9:34. 10.3390/jcm9010034 31877865 PMC7019226

[B10] Fu J. Liu Q. Du Y. Zhu Y. Sun C. Lin H. (2020). Age- and sex-specific prevalence and modifiable risk factors of mild cognitive impairment among older adults in China: a population-based observational study. *Front. Aging Neurosci.* 12:578742. 10.3389/fnagi.2020.578742 33192471 PMC7662098

[B11] Gorelick P. B. Scuteri A. Black S. E. Decarli C. Greenberg S. M. Iadecola C. (2011). Vascular contributions to cognitive impairment and dementia: a statement for healthcare professionals from the American heart association/American stroke association. *Stroke* 42 2672–2713. 10.1161/STR.0b013e3182299496 21778438 PMC3778669

[B12] Gottesman R. F. Albert M. S. Alonso A. Coker L. H. Coresh J. Davis S. M. (2017). Associations between midlife vascular risk factors and 25-year incident dementia in the atherosclerosis risk in communities (ARIC) cohort. *JAMA Neurol.* 74 1246–1254. 10.1001/jamaneurol.2017.1658 28783817 PMC5710244

[B13] Hajjar I. Hayek S. S. Goldstein F. C. Martin G. Jones D. P. Quyyumi A. (2018). Oxidative stress predicts cognitive decline with aging in healthy adults: an observational study. *J. Neuroinflammation* 15:17. 10.1186/s12974-017-1026-z 29338747 PMC5771063

[B14] Hamilton L. D. Thomas E. Almuklass A. M. Enoka R. M. (2017). A framework for identifying the adaptations responsible for differences in pegboard times between middle-aged and older adults. *Exp. Gerontol.* 97 9–16. 10.1016/j.exger.2017.07.003 28688836 PMC5591777

[B15] Hernán M. A. Robins J. M. (2020). *Causal Inference: What If.* Boca Raton, FL: Chapman & Hall/CRC.

[B16] Iadecola C. Gottesman R. F. (2019). Neurovascular and cognitive dysfunction in hypertension: epidemiology, pathobiology, and treatment. *Circ. Res.* 124 1025–1044. 10.1161/CIRCRESAHA.118.313260 30920929 PMC6527115

[B17] Kalibala J. Pechère-Bertschi A. Desmeules J. (2020). Gender differences in cardiovascular pharmacotherapy—the example of hypertension: a mini review. *Front. Pharmacol.* 11:564. 10.3389/fphar.2020.00564 32435193 PMC7218117

[B18] Khera A. McGuire D. K. Murphy S. A. Stanek H. G. Das S. R. Vongpatanasin W. (2005). Race and gender differences in C-reactive protein levels. *J. Am. Coll. Cardiol.* 46 464–469. 10.1016/j.jacc.2005.04.051 16053959

[B19] Kissler J. Assadollahi R. Herbert C. (2006). Emotional and semantic networks in visual word processing: insights from ERP studies. *Prog. Brain Res.* 156 147–183. 10.1016/S0079-6123(06)56008-X 17015079

[B20] Kluger A. Gianutsos J. G. Golomb J. Ferris S. H. George A. E. Franssen E. (1997). Patterns of motor impairment in normal aging, mild cognitive decline, and early Alzheimer’ disease. *J. Gerontol. Ser. B* 52B 28–39. 10.1093/geronb/52B.1.P28 9008673

[B21] Lakoski S. G. Cushman M. Criqui M. Rundek T. Blumenthal R. S. D’Agostino R. B. (2006). Gender and C-reactive protein: data from the Multiethnic Study of Atherosclerosis (MESA) cohort. *Am. Heart J.* 152 593–598. 10.1016/j.ahj.2006.02.015 16923436

[B22] Lefort M. Neufcourt L. Pannier B. Vaïsse B. Bayat S. Grimaud O. (2018). Sex differences in adherence to antihypertensive treatment in patients aged above 55: the French League Against Hypertension Survey (FLAHS). *J. Clin. Hypertens. Greenwich Conn* 20 1496–1503. 10.1111/jch.13387 30238630 PMC8030937

[B23] Lerner D. J. Kannel W. B. (1986). Patterns of coronary heart disease morbidity and mortality in the sexes: a 26-year follow-up of the Framingham population. *Am. Heart J.* 111 383–390. 10.1016/0002-8703(86)90155-9 3946178

[B24] Levine D. A. Gross A. L. Briceño E. M. Tilton N. Giordani B. J. Sussman J. B. (2021). Sex differences in cognitive decline among US adults. *JAMA Netw. Open* 4:e210169. 10.1001/jamanetworkopen.2021.0169 33630089 PMC7907956

[B25] Livingston G. Huntley J. Sommerlad A. Ames D. Ballard C. Banerjee S. (2020). Dementia prevention, intervention, and care: 2020 report of the Lancet Commission. *Lancet Lond. Engl.* 396 413–446. 10.1016/S0140-6736(20)30367-6 32738937 PMC7392084

[B26] Long J. A. (2019). *interactions: Comprehensive, User-Friendly Toolkit for Probing Interactions. R package version 1.1.0.* Available online at: https://cran.r-project.org/package=interactions

[B27] Luck T. Pabst A. Rodriguez F. S. Schroeter M. L. Witte V. Hinz A. (2018). Age-, sex-, and education-specific norms for an extended CERAD Neuropsychological Assessment Battery—Results from the population-based LIFE-Adult-Study. *Neuropsychology* 32 461–475. 10.1037/neu0000440 29517259

[B28] Marrocco J. McEwen B. S. (2016). Sex in the brain: hormones and sex differences. *Dialogues Clin. Neurosci.* 18 373–383. 10.31887/DCNS.2016.18.4/jmarrocco 28179809 PMC5286723

[B29] McCarrey A. C. An Y. Kitner-Triolo M. H. Ferrucci L. Resnick S. M. (2016). Sex differences in cognitive trajectories in clinically normal older adults. *Psychol. Aging* 31 166–175. 10.1037/pag0000070 26796792 PMC4783196

[B30] McEwen B. S. Milner T. A. (2017). Understanding the broad influence of sex hormones and sex differences in the brain. *J. Neurosci. Res.* 95 24–39. 10.1002/jnr.23809 27870427 PMC5120618

[B31] Monette M. C. E. Baird A. Jackson D. L. (2014). A meta-analysis of cognitive functioning in nondemented adults with type 2 diabetes mellitus. *Can. J. Diabetes* 38 401–408. 10.1016/j.jcjd.2014.01.014 24933107

[B32] Morris J. C. Heyman A. Mohs R. C. Hughes J. P. van Belle G. Fillenbaum G. (1989). The consortium to establish a registry for Alzheimer’s Disease (CERAD). Part I. Clinical and neuropsychological assessment of Alzheimer’s disease. *Neurology* 39 1159–1165. 10.1212/wnl.39.9.1159 2771064

[B33] Nichols E. S. Wild C. J. Owen A. M. Soddu A. (2021). Cognition across the lifespan: investigating age, sex, and other sociodemographic influences. *Behav. Sci.* 11:51. 10.3390/bs11040051 33924660 PMC8070049

[B34] Peters M. Servos P. Day R. (1990). Marked sex differences on a fine motor skill task disappear when finger size is used as covariate. *J. Appl. Psychol.* 75 87–90. 10.1037/0021-9010.75.1.87 2307635

[B35] R Core Team (2021). *R: A Language and Environment for Statistical Computing.* Vienna: R Foundation for Statistical Computing.

[B36] Radloff L. S. (1977). The CES-D scale: a self-report depression scale for research in the general population. *Appl. Psychol. Meas.* 1 385–401. 10.1177/014662167700100306 26918431

[B37] Reas E. T. Laughlin G. A. Bergstrom J. Kritz-Silverstein D. Barrett-Connor E. McEvoy L. K. (2017). Effects of sex and education on cognitive change over a 27-year period in older adults: the rancho Bernardo study. *Am. J. Geriatr. Psychiatry* 25 889–899. 10.1016/j.jagp.2017.03.008 28433548 PMC5522346

[B38] Reitan R. M. (1992). *Trail Making Test: Manual for Administration and Scoring.* Tucson, AZ: Reitan Neuropsychology Laboratory.

[B39] Revelle W. (2021). *psych: Procedures for Psychological, Psychometric, and Personality Research.* Evanston, IL: Northwestern University.

[B40] Rouanet A. Avila-Rieger J. Dugravot A. Lespinasse J. Stuckwisch R. Merrick R. (2021). How selection over time contributes to the inconsistency of the association between sex/gender and cognitive decline across cognitive aging cohorts. *Am. J. Epidemiol.* 191, 441–452. 10.1093/aje/kwab227 34521111 PMC9214252

[B41] RStudio Team (2021). *RStudio: Integrated Development Environment for R.* Boston, MA: RStudio, PBC.

[B42] Salthouse T. A. (2019). Trajectories of normal cognitive aging. *Psychol. Aging* 34 17–24. 10.1037/pag0000288 30211596 PMC6367038

[B43] Schmidt S. L. Oliveira R. M. Rocha F. R. Abreu-Villaca Y. (2000). Influences of handedness and gender on the grooved pegboard test. *Brain Cogn.* 44 445–454. 10.1006/brcg.1999.1204 11104536

[B44] Schneider G. Köhnke C. Teismann H. Berger K. (2021). Childhood trauma and personality explain more variance in depression scores than sociodemographic and lifestyle factors - Results from the BiDirect Study. *J. Psychosom. Res.* 147:110513. 10.1016/j.jpsychores.2021.110513 34022671

[B45] Seaman S. R. White I. R. (2013). Review of inverse probability weighting for dealing with missing data. *Stat. Methods Med. Res.* 22 278–295. 10.1177/0962280210395740 21220355

[B46] Seedat S. Scott K. M. Angermeyer M. C. Berglund P. Bromet E. J. Brugha T. S. (2009). Cross-national associations between gender and mental disorders in the World Health Organization World Mental Health Surveys. *Arch. Gen. Psychiatry* 66 785–795. 10.1001/archgenpsychiatry.2009.36 19581570 PMC2810067

[B47] Sivagnanasunderam M. Gonzalez D. A. Bryden P. J. Young G. Forsyth A. Roy E. A. (2015). Handedness throughout the lifespan: cross-sectional view on sex differences as asymmetries change. *Front. Psychol.* 5:1556. 10.3389/fpsyg.2014.01556 25642200 PMC4293916

[B48] Stroop J. R. (1935). Studies of interference in serial verbal reactions. *J. Exp. Psychol.* 18:643. 10.1037/h0054651

[B49] Teismann H. Wersching H. Nagel M. Arolt V. Heindel W. Baune B. T. (2014). Establishing the bidirectional relationship between depression and subclinical arteriosclerosis–rationale, design, and characteristics of the BiDirect Study. *BMC Psychiatry* 14:174. 10.1186/1471-244X-14-174 24924233 PMC4065391

[B50] Teuber A. Sundermann B. Kugel H. Schwindt W. Heindel W. Minnerup J. (2017). MR imaging of the brain in large cohort studies: feasibility report of the population- and patient-based BiDirect study. *Eur. Radiol.* 27 231–238. 10.1007/s00330-016-4303-9 27059857

[B51] Tiffin J. Asher E. J. (1948). The Purdue pegboard; norms and studies of reliability and validity. *J. Appl. Psychol.* 32 234–247. 10.1037/h0061266 18867059

[B52] Tombaugh T. N. Kozak J. Rees L. (1999). Normative data stratified by age and education for two measures of verbal fluency: FAS and animal naming. *Arch. Clin. Neuropsychol. Off. J. Natl. Acad. Neuropsychol.* 14 167–177. 14590600

[B53] Van der Elst W. Van Boxtel M. P. J. Van Breukelen G. J. P. Jolles J. (2006). The stroop color-word test: influence of age, sex, and education; and normative data for a large sample across the adult age range. *Assessment* 13 62–79. 10.1177/1073191105283427 16443719

[B54] van Zutphen E. M. Rijnhart J. J. M. Rhebergen D. Muller M. Huisman M. Beekman A. (2021). Do cardiovascular risk factors and cardiovascular disease explain sex differences in cognitive functioning in old age? *J. Alzheimers Dis.* 80 1643–1655. 10.3233/JAD-201173 33720886 PMC8150475

[B55] Vintimilla R. Hall J. Johnson L. O’Bryant S. (2019). The relationship of CRP and cognition in cognitively normal older Mexican Americans: a cross-sectional study of the HABLE cohort. *Medicine (Baltimore)* 98:e15605. 31083252 10.1097/MD.0000000000015605 PMC6531144

[B56] Volgman A. S. Bairey Merz C. N. Aggarwal N. T. Bittner V. Bunch T. J. Gorelick P. B. (2019). Sex differences in cardiovascular disease and cognitive impairment: another health disparity for women? *J. Am. Heart Assoc.* 8:e013154. 10.1161/JAHA.119.013154 31549581 PMC6806032

[B57] Walker K. A. Gottesman R. F. Wu A. Knopman D. S. Gross A. L. Mosley T. H. (2019). Systemic inflammation during midlife and cognitive change over 20 years. *Neurology* 92 e1256–e1267. 10.1212/WNL.0000000000007094 30760633 PMC6511107

[B58] Zheng F. Xie W. (2018). High-sensitivity C-reactive protein and cognitive decline: the English Longitudinal Study of Ageing. *Psychol. Med.* 48 1381–1389. 10.1017/S0033291717003130 29108529

