# Supplementary material for: The Relative Importance of Vascular Risk Factors on Early Cognitive Aging Varies Only Slightly Between Men and Women
Source: Front Aging Neurosci. 2022 Mar 28;14:804842. doi: 10.3389/fnagi.2022.804842 (PMC8996124; doi:10.3389/fnagi.2022.804842)
Supplement: Supplementary file 1 [file Data_Sheet_1.docx]

**Supplementary Material**

**Table S1a.** Sex-specific effects of risk factors on memory (Word List Test) and fine motor skills (Purdue Pegboard Test). Alternative definition of arterial hypertension. Results from linear mixed models stratified by sex.

|  | Word List | | Pegboard | |
| --- | --- | --- | --- | --- |
|  | men | women | men | women |
|  | $\hat{\beta}$ (95% CI) | $\hat{\beta}$ (95% CI) | $\hat{\beta}$ (95% CI) | $\hat{\beta}$ (95% CI) |
| Diabetes (Ref: no) |  |  |  |  |
| >0-7 years | 0.11 | 0.01 | -0.25 | 0.05 |
|  | (-0.22, 0.44) | (-0.30, 0.32) | (-0.60, 0.10) | (-0.29, 0.39) |
| >7 years | 0.03 | 0.08 | -0.90^***^ | -0.31 |
|  | (-0.37, 0.44) | (-0.31, 0.47) | (-1.32, -0.47) | (-0.75, 0.12) |
| Hypertension (Ref: no) |  |  |  |  |
| >0-10 years | -0.03 | -0.07 | 0.05 | -0.04 |
|  | (-0.16, 0.10) | (-0.20, 0.06) | (-0.09, 0.20) | (-0.18, 0.10) |
| >10 years | 0.01 | -0.17 | 0.26^*^ | 0.01 |
|  | (-0.19, 0.21) | (-0.37, 0.04) | (0.05, 0.47) | (-0.21, 0.23) |
| Smoking (Ref.: Never) |  |  |  |  |
| Former | -0.10 | 0.05 | -0.17^*^ | -0.22^**^ |
|  | (-0.25, 0.05) | (-0.08, 0.18) | (-0.33, -0.01) | (-0.36, -0.08) |
| Current | -0.12 | -0.01 | -0.35^***^ | -0.30^***^ |
|  | (-0.29, 0.06) | (-0.18, 0.15) | (-0.53, -0.16) | (-0.48, -0.13) |
| Obesity (Ref: No,) |  |  |  |  |
| Yes | -0.04 | -0.06 | -0.09 | -0.11 |
|  | (-0.19, 0.11) | (-0.20, 0.09) | (-0.25, 0.07) | (-0.27, 0.04) |
| Marginal R-squared | 0.26 | 0.26 | 0.29 | 0.24 |
| Conditional R-squared | 0.71 | 0.67 | 0.69 | 0.65 |
|  | ^*^p<0.05, ^**^p<0.01, ^***^p<0.001 | | | |

Weighted linear mixed models with 888 examinations from 373 men and 1023 examinations from 425 women, respectively, adjusted for education, CES-D score, age (natural spline, df=2), number of study participations and, for memory, the interaction of age and number of study participations. Models for Pegboard are additionally adjusted for body height.

**Table S1b.** Sex-specific effects of risk factors on CWIT (interference time), TMT A, TMT B, the Word Fluency Test and the global score. Alternative definition of arterial hypertension. Results from linear mixed models stratified by sex.

|  | CWIT (interference time) | | TMT A | | TMT B | | Word Fluency Test | | Global Score | |
| --- | --- | --- | --- | --- | --- | --- | --- | --- | --- | --- |
|  | men | women | men | women | men | women | men | women | men | women |
|  | $\hat{\beta}$ (95% CI) | $\hat{\beta}$ (95% CI) | $\hat{\beta}$ (95% CI) | $\hat{\beta}$ (95% CI) | $\hat{\beta}$ (95% CI) | $\hat{\beta}$ (95% CI) | $\hat{\beta}$ (95% CI) | $\hat{\beta}$ (95% CI) | $\hat{\beta}$ (95% CI) | $\hat{\beta}$ (95% CI) |
| Study participation (Ref: First) |  |  |  |  |  |  |  |  |  |  |
| Second | 0.26^***^ | 0.17^***^ | 0.02 | 0.05 | 0.01 | 0.08 | 0.05 | 0.19^***^ | 0.16^***^ | 0.22^***^ |
|  | (0.16, 0.36) | (0.08, 0.27) | (-0.09, 0.12) | (-0.05, 0.14) | (-0.08, 0.10) | (-0.003, 0.17) | (-0.05, 0.15) | (0.10, 0.29) | (0.09, 0.23) | (0.16, 0.29) |
| Third | 0.37^***^ | 0.36^***^ | 0.15^*^ | 0.14^*^ | 0.04 | 0.14^*^ | 0.01 | 0.25^***^ | 0.24^***^ | 0.38^***^ |
|  | (0.25, 0.49) | (0.25, 0.48) | (0.03, 0.27) | (0.02, 0.26) | (-0.08, 0.15) | (0.03, 0.25) | (-0.11, 0.14) | (0.13, 0.36) | (0.15, 0.33) | (0.30, 0.46) |
| Diabetes (Ref: no) |  |  |  |  |  |  |  |  |  |  |
| >0-7 years | -0.09 | -0.12 | -0.45^*^ | 0.07 | -0.07 | -0.02 | -0.17 | 0.08 | -0.26 | 0.13 |
|  | (-0.48, 0.30) | (-0.49, 0.26) | (-0.85, -0.05) | (-0.32, 0.45) | (-0.44, 0.30) | (-0.38, 0.34) | (-0.58, 0.24) | (-0.32, 0.47) | (-0.56, 0.05) | (-0.15, 0.42) |
| >7 years | -0.72^**^ | -0.23 | -0.62^**^ | -0.05 | -0.44 | -0.45 | -0.43 | -0.31 | -0.71^***^ | -0.32 |
|  | (-1.19, -0.25) | (-0.70, 0.24) | (-1.09, -0.15) | (-0.54, 0.44) | (-0.89, 0.02) | (-0.92, 0.01) | (-0.93, 0.07) | (-0.81, 0.20) | (-1.12, -0.30) | (-0.71, 0.07) |
| Hypertension (Ref: no) |  |  |  |  |  |  |  |  |  |  |
| >0-10 years | -0.01 | 0.11 | 0.12 | -0.05 | 0.06 | -0.05 | -0.09 | -0.16 | 0.08 | -0.07 |
|  | (-0.16, 0.15) | (-0.05, 0.26) | (-0.04, 0.28) | (-0.21, 0.11) | (-0.09, 0.21) | (-0.20, 0.10) | (-0.25, 0.08) | (-0.32, 0.01) | (-0.05, 0.20) | (-0.19, 0.05) |
| >10 years | -0.09 | 0.02 | 0.04 | -0.02 | 0.16 | 0.02 | 0.05 | 0.07 | 0.13 | -0.05 |
|  | (-0.33, 0.14) | (-0.22, 0.26) | (-0.20, 0.27) | (-0.26, 0.23) | (-0.06, 0.38) | (-0.22, 0.26) | (-0.20, 0.30) | (-0.19, 0.32) | (-0.08, 0.33) | (-0.25, 0.15) |
| Smoking (Ref.: Never) |  |  |  |  |  |  |  |  |  |  |
| Former | -0.10 | -0.09 | -0.02 | -0.01 | -0.09 | 0.01 | -0.06 | 0.02 | -0.12 | -0.07 |
|  | (-0.28, 0.07) | (-0.24, 0.07) | (-0.19, 0.16) | (-0.17, 0.15) | (-0.26, 0.08) | (-0.15, 0.17) | (-0.25, 0.12) | (-0.14, 0.19) | (-0.27, 0.04) | (-0.21, 0.06) |
| Current | 0.04 | -0.19 | -0.09 | -0.14 | -0.17 | -0.15 | -0.22^*^ | -0.04 | -0.16 | -0.20^*^ |
|  | (-0.17, 0.25) | (-0.38, 0.001) | (-0.29, 0.12) | (-0.34, 0.06) | (-0.37, 0.02) | (-0.35, 0.04) | (-0.44, -0.003) | (-0.25, 0.16) | (-0.34, 0.02) | (-0.37, -0.04) |
| Obesity (Ref: No) |  |  |  |  |  |  |  |  |  |  |
| Yes | -0.08 | -0.17 | -0.06 | -0.04 | -0.16 | -0.04 | -0.03 | -0.10 | -0.12 | -0.15^*^ |
|  | (-0.25, 0.10) | (-0.34, 0.002) | (-0.24, 0.12) | (-0.21, 0.14) | (-0.33, 0.003) | (-0.21, 0.13) | (-0.21, 0.16) | (-0.28, 0.09) | (-0.26, 0.03) | (-0.29, -0.01) |
| Marginal R-squared | 0.20 | 0.19 | 0.19 | 0.13 | 0.22 | 0.18 | 0.09 | 0.16 | 0.38 | 0.39 |
| Conditional R-squared | 0.66 | 0.58 | 0.62 | 0.56 | 0.70 | 0.66 | 0.63 | 0.60 | 0.86 | 0.83 |
|  | ^*^p<0.05, ^**^p<0.01, ^***^p<0.001 | | | | | | | | | |

Weighted linear mixed models with 888 examinations from 373 men and 1023 examinations from 425 women, respectively, adjusted for age (natural spline, df=2), education and CES-D score.
